# Sustainability of Biosimilars in Europe: A Delphi Panel Consensus with Systematic Literature Review

**DOI:** 10.3390/ph13110400

**Published:** 2020-11-17

**Authors:** Arnold G. Vulto, Jackie Vanderpuye-Orgle, Martin van der Graaff, Steven R. A. Simoens, Lorenzo Dagna, Richard Macaulay, Beenish Majeed, Jeffrey Lemay, Jane Hippenmeyer, Sebastian Gonzalez-McQuire

**Affiliations:** 1 Hospital Pharmacy, Erasmus University Medical Center, NL-3015 CN Rotterdam, The Netherlands; a.vulto@gmail.com; 2 Department of Pharmaceutical and Pharmacological Sciences, KU Leuven, 3000 Leuven, Belgium; steven.simoens@kuleuven.be; 3 Access Consulting, Parexel International, Billerica, MA 01821, USA; 4 Ex-National Health Care Institute, Zorginstituut Nederland (ZIN), NL-1110 AH Diemen, The Netherlands; m.vandergraaff@ziggo.nl; 5 IRCCS San Raffaele Scientific Institute, Universita Vita-Salute San Raffaele, 20132 Milan, Italy; dagna.lorenzo@unisr.it; 6 Access Consulting, Parexel International, Uxbridge UB8 ILZ, UK; Richard.Macaulay@parexel.com (R.M.); Beenish.Majeed@parexel.com (B.M.); 7 Amgen Inc., Thousand Oaks, CA 91320, USA; jlemay@amgen.com; 8 Amgen Inc. Europe GmbH, CH-6343 Rotkreuz, Switzerland; jhippenm@amgen.com (J.H.); sebgonza@amgen.com (S.G.-M.)

**Keywords:** biosimilar market, biosimilar/supply and distribution, biosimilar sustainability, Delphi technique

## Abstract

Introduction: Biosimilars have the potential to enhance the sustainability of evolving health care systems. A sustainable biosimilars market requires all stakeholders to balance competition and supply chain security. However, there is significant variation in the policies for pricing, procurement, and use of biosimilars in the European Union. A modified Delphi process was conducted to achieve expert consensus on biosimilar market sustainability in Europe. Methods: The priorities of 11 stakeholders were explored in three stages: a brainstorming stage supported by a systematic literature review (SLR) and key materials identified by the participants; development and review of statements derived during brainstorming; and a facilitated roundtable discussion. Results: Participants argued that a sustainable biosimilar market must deliver tangible and transparent benefits to the health care system, while meeting the needs of all stakeholders. Key drivers of biosimilar market sustainability included: (i) competition is more effective than regulation; (ii) there should be incentives to ensure industry investment in biosimilar development and innovation; (iii) procurement processes must avoid monopolies and minimize market disruption; and (iv) principles for procurement should be defined by all stakeholders. However, findings from the SLR were limited, with significant gaps on the impact of different tender models on supply risks, savings, and sustainability. Conclusions: A sustainable biosimilar market means that all stakeholders benefit from appropriate and reliable access to biological therapies. Failure to care for biosimilar market sustainability may impoverish biosimilar development and offerings, eventually leading to increased cost for health care systems and patients, with fewer resources for innovation.

## 1. Introduction

The global biosimilars market was valued at $4.5 billion in 2019 and is expected to reach $23.6 billion by 2024; this is an estimated growth rate of 39.4%, with most of this growth occurring in Europe [1,2]. Such a rapid acceleration in the biosimilars market may result in numerous challenges, and it is important to support a thoughtful deployment of biosimilars. This will provide an opportunity for sustainability of global health care budgets and evolving health care systems [3,4].

At present, the European Medicines Agency (EMA) defines a “biosimilar” as “a biological medicinal product that contains a version of the active substance of an already authorized original biological medicinal product (reference medicinal product)” for which “similarity to the reference medicinal product in terms of quality characteristics, biological activity, safety, and efficacy based on a comprehensive comparability exercise needs to be established” [5,6]. Manufacturers in both the United States and Europe are required to demonstrate that the proposed biosimilar and its reference product are highly similar and have no clinically meaningful differences [5,7].

Thus, biosimilars are manufactured following the same strict standards of quality, safety, and efficacy observed for the reference product [5,7]; this is reflected in the development cost, which ranges from $100 to 300 million [8]. Biosimilars can broaden product choice and have the potential to reduce prices, whilst continuing to support a high standard of patient care [9]. In the United States, potential cost saving from switching from originator biologics to biosimilars is projected to be between $40 and 250 billion by 2025, and in Europe, cost savings are already estimated to be more than €10 billion [2,10,11,12].

Many organizations representing physicians, pharmacists, and patients across Europe support the use of biosimilars [3,4,13,14,15], and have issued position papers outlining best practices for their use. However, biosimilar markets are still evolving, and there are marked differences between policies and practices across European countries [16,17,18]. For example, some payer bodies have implemented single winner tender-based systems. While this can secure significant short-term payer savings, such systems risk locking out many biosimilar manufacturers, and may limit the number of competing manufacturers in the medium term. In addition, single-manufacturer tenders can place a lot of risk on the supply chain and, potentially, on patient access. Therefore, systems need to be set up to ensure that long-term savings are realized for payers and sufficient manufacturer incentives are in place to sustain multiplayer competition. Further, the notion of biosimilar sustainability is currently inconsistently and poorly defined, and there is a lack of awareness on the vulnerability of the current system. Previous analyses of the biosimilars market have concluded that there is a need to improve sustainability, and several areas have been identified for further research to develop a coherent long-term vision of sustainability. These include safeguarding the interests of patients, maintaining physician autonomy and patient choice, effective purchasing/pricing and reimbursement strategies, good pharmacovigilance practices, and healthy levels of competition to ensure consistent supply of a range of high-quality products [11,19,20].

To consider these issues and examine biosimilar market sustainability in more detail, we conducted a systematic literature review (SLR) and Delphi panel discussion to: (i) establish a multistakeholder definition of biosimilar market sustainability; (ii) further identify components of a sustainable biosimilar market; and (iii) identify drivers and risks of a sustainable biosimilar market.

## 2. Methods

### 2.1. Design

**Figure 1:**
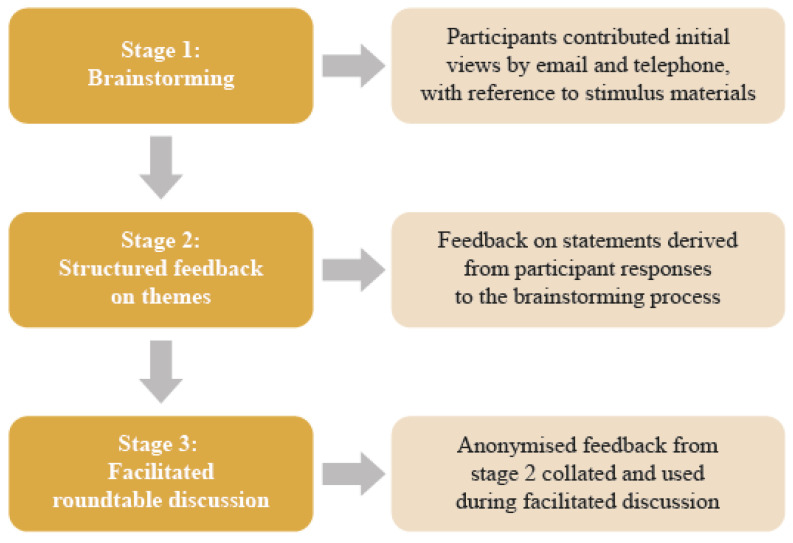
Modified Delphi process.

The modified Delphi process is a commonly published approach to generate discussion around topics without consensus and is an effective way to start dealing with complex multifactorial challenges [21]. A modified Delphi process, involving 11 key opinion leaders representing various sectors of the health care system in Europe, was conducted between September and November 2019. Participating stakeholders comprised one patient advocate, two physicians, two hospital pharmacists, two procurement pharmacists, one national payer, two policy advisors, and one manufacturer from across Europe. The modified Delphi process was based on a published approach, [22] and consisted of brainstorming, structured feedback, and a facilitated roundtable discussion (Figure 1).

### 2.2. Procedure

The Delphi process was initiated by multiple stages of brainstorming, in which participants contributed their initial views by email and telephone using the questionnaire shown in Appendix A. Participants were provided with stimulus materials identified by an SLR and they were also asked to identify any key papers to support their feedback. The SLR is briefly described in this paper, but it is published elsewhere [18].

The SLR was conducted using EMBASE, MEDLINE, and grey literature searches. The searches were conducted using recent evidence (from 2008 to 2019) to capture all key biosimilar publications after their introduction in Europe in 2006. Only publications in English were included. Search methods were based on recommendations from the Cochrane Handbook [23] and the Centre for Reviews and Dissemination [24]. The SLR identified materials relating to major economies in Europe and answered three predefined key questions on the: (Q1) frequency, causes, and consequences of shortages of reference product biologics and biosimilars; (Q2) costs (direct and indirect costs, resource utilization, and external costs) and impacts resulting from switching patients between biosimilar products; and (Q3) causation between tendering, market concentration, drug shortages, and achievement of savings, and the implications of tender models for supply risk (reliability) and sustainability of competitive biosimilar markets.

In an initial screening phase, one reviewer identified relevant titles and abstracts from among all retrieved records; in a second screening stage, one reviewer re-evaluated each selected publication in a full-text review. In both stages, a second reviewer was consulted in cases of uncertainty, and consensus between the two was reached. Data extraction was performed by two reviewers, with one extracting the data and the second checking the data against the original publication. Any discrepancies were resolved through discussion or through the intervention of a third reviewer. The SLR flow chart is shown in Appendix B. The findings of the SLR were provided to the panel during the brainstorming stage; participants reviewed the material (amendments were allowed) and provided their ideas. The feedback was discussed with each participant via telephone to ensure that it was interpreted correctly, and the finalized brainstorming responses were collected via email. The amended stimulus material is shown in Appendix C [3,4,16,17,18,25,26,27]. These references and the brainstorming responses were used to develop the themes and statements for the second stage.

In the second stage of the Delphi process, the brainstorming responses and evidence extracted from the stimulus materials were converted into themes and statements (Appendix D), using standard primary research methodology. Feedback was sought on: (i) the components for a definition of biosimilar market sustainability and (ii) drivers and risks to achieving sustainability. Participants were asked to indicate on a Likert scale (from “strongly disagree” to “strongly agree”) their level of agreement with each theme and statement, and how strongly they felt the evidence supported each theme and statement. Each participant had 1 week to provide their responses.

Stage 3 was a facilitated roundtable discussion that aimed to derive a multistakeholder definition of sustainability and achieve consensus on the components of a sustainable biosimilar market. First, the participants were presented with a definition of sustainability (derived from feedback supplied in stage 2) and were then asked to provide feedback on the definition, stating their level of agreement (“strongly disagree” to “strongly agree”), and providing any revisions they wished to make in free text. Based on the responses, the initial definition was revised and presented to participants for comment and final agreement at the end of the roundtable discussion. Participants were then presented with eight statements on the components of a sustainable biosimilar market and on drivers and risks to sustainability (derived from feedback supplied in stage 2). Participants were asked to provide individual feedback regarding how much they agreed with each statement, how important each statement was to them, and free-text suggestions on how to rephrase each statement so that it would align better with their views. The purpose of the discussions on each statement was to explore areas of agreement and disagreement between stakeholder groups. Where groups agreed, consensus was noted; however, the process could cease if stakeholder views remained divergent.

## 3. Results

### 3.1. Delphi Panel Consensus

#### 3.1.1. A Multistakeholder Definition of Biosimilar Market Sustainability

The multistakeholder consensus definition of a sustainable biosimilars market is provided in Box 1. After much deliberation, this definition was agreed upon by all participants; however, different stakeholder groups emphasized different priorities within this definition. Patients wanted to be well-informed, physicians wanted biosimilar-related savings reinvested, pharmacists/manufacturers emphasized quality, and payers/policy advisers focused on mechanisms (e.g., competition) to lower prices. These differing priorities were not considered to be mutually exclusive, and all participants considered it important to incorporate the perspectives of all stakeholders into the definition of a sustainable biosimilar market in Europe.

Box 1 **A multistakeholder consensus definition of a sustainable biosimilar market.**

- A sustainable biosimilar market means that…
  “All stakeholders, including patients, benefit from appropriate and reliable access to biological therapies. Competition leads to a long-term predictable price level, without compromising quality, while delivering savings that may be reinvested.”

#### 3.1.2. Components of a Sustainable Biosimilar Market

Participants agreed that a sustainable biosimilar market: (i) must deliver tangible and transparent benefits to the health care system; (ii) must address the needs of all stakeholders; and (iii) requires collaboration between stakeholders. The level of consensus achieved on these key points is summarized in Box 2 and Table 1.

Box 2 **Consensus on components of a sustainable biosimilar market.**

A sustainable biosimilar market must:

- Deliver tangible and transparent benefits to the health care system, while
- Addressing the needs of all stakeholders

This requires collaboration between stakeholders.

In brief, participants strongly agreed that biosimilars have the potential to promote competition among biologic options and reduce treatment costs. However, there was a need to identify and minimize transition and disruption costs when switching to a biosimilar or between biosimilars to improve savings associated with these products further. These savings should be tangible (i.e., measurable) and reinvested in health care or other public services where possible. This could include budget deficits and funding of innovative therapies; biosimilars have the potential to expand access [8]. Transparency regarding reinvestment was regarded as another important motivator for physicians and patients to use biosimilars. Minimizing transition costs could be achieved by identifying key differences between therapeutic areas and clinical settings. For example, oncology treatments usually follow a short, defined treatment course reducing the need for switch, whereas rheumatoid arthritis treatments may be chronic with multiple use of biosimilars and combinations. Clear guidance (policies and practices) from regulators and clinical organizations, such as the EMA, regarding biosimilar transition is warranted with the need for real-world evidence based on biosimilars that physicians can effectively communicate to patients to avoid any negative perceptions. Collaboration between stakeholders would help enable any guidance to be consistent, more comprehensive, and more easily communicated.

#### 3.1.3. Drivers and Risks of a Sustainable Biosimilar Market (Competition and Incentives)

The consensus achieved by participants regarding drivers and risks of a sustainable biosimilar market is summarized in Box 3 and Table 2. Points of consensus were formulated as follows: (i) competition is a more effective mechanism to achieve a long-term predictable price level than regulation; (ii) there needs to be incentives for investment in future biosimilars; and (iii) government and pricing bodies need to drive incentives.

Box 3 **Consensus on drivers of and risks to a sustainable biosimilar market.**

- Competition is a more effective mechanism to achieve a long-term predictable price level, compared to regulation
- There needs to be incentives for industry investment in future biosimilars
- Government and pricing bodies need to drive incentives
- Procurement processes should avoid monopolies and minimize patient discomfort and disruption to the health care system
- The principles for procurement should be defined by all stakeholders.

For key market drivers, participants agreed that competition generated by the introduction of biosimilars has been effective in reducing prices for biological therapies in Europe [28,29]. Participants also agreed that the price expectations of decision makers must reflect market opportunity. This was illustrated by the case of adalimumab biosimilars, the entry of which into the market in 2018 triggered almost immediate and substantial discounting. However, adalimumab was used in a large patient population and had achieved extremely high revenues prior to biosimilar entry, making it a very attractive target for biosimilar manufacturers. Consequently, the price levels achieved by adalimumab biosimilars might not be repeated in other biosimilar products, especially those with orphan status. Incentives driven by governments and pricing bodies (such as limits on tender) were also identified as key drivers for future market; these incentives could include procurement design, including contract length, a cap on the number of manufacturers selected, and introduction of geographical divisions (national vs. regional vs. local).

For key market risks, there was agreement that there is a need for better indicators than those currently available (e.g., the number of biosimilar manufacturers and manufacturing sites) to warn of potential de facto monopoly [30]. Participants agreed that the emergence of monopolies could lead to higher price levels and/or enhanced supply risks (such as poor quality), or supply shortages (e.g., limited production capabilities and poor distribution channels) for biosimilars. This risk also exists for generics, but it would be greater for biosimilars due to the lengthier development and market entry processes, and the much longer lead time in manufacturing (1 year or more). Participants felt that there was a need for more research to identify prospective indicators of market performance; these should be based on a thorough understanding of the role that procurement level (national vs. subnational (procurement is described below)), market size, number of awarded contracts (and market share awarded), and tender criteria may play in ensuring markets perform well. Unfortunately, published evidence on indicator performance or biosimilar supply risks and shortages are scarce making generalizability difficult, but also highlighting the need for establishing validated approaches to long-term quantification of these frameworks.

#### 3.1.4. Drivers and Risks of a Sustainable Biosimilar Market (Procurement Processes)

Issues surrounding procurement processes are summarized in Box 3 and Table 3. Participants agreed that procurement processes should avoid monopolies and minimize patient and health care system disruption, and the principles for procurement should be agreed by all stakeholders. The participants also identified two main goals of procurement design from a multistakeholder perspective. The first goal was to prevent predatory behavior by considering factors in selection criteria other than price or aggressive price discounting; these could include differentiation based on formulation and quality attributes, or stock and distribution channels. The second goal was to minimize disruptions to patient care based on the needs of individual therapy areas, perhaps by setting a contract duration that is proportional to the duration of treatment. Given the potential implications of procurement policies for all stakeholders, participants agreed that all stakeholders should have a voice in setting procurement policies. Participants agreed that there cannot be a “one size fits all” approach to procurement, as the structure and characteristics of health care systems vary; however, procurement policies should be consistent, guided by a common set of principles, and abide with European Union rules on tendering. Participants also advised that biosimilar procurement must be managed carefully over the product lifecycle to preserve competition and promote new investment in biosimilar development.

### 3.2. Key Findings from the SLR

A total of 36 studies were identified in the SLR (Appendix B). Nine publications were identified that discussed (Q1). However, these were too limited to provide any comprehensive evidence and demonstrate the lack of a consistent, comprehensive database of medicine shortages in Europe. Nineteen publications addressed (Q2). None of these reported switching between biosimilars; rather, all considered switches from a reference product to a biosimilar. Nine publications focused on (Q3). These offered insufficient evidence from which to reach generalized conclusions about the effects of different tender models on the outcomes of interest. However, one policy paper concluded that barriers to entry, including the use of single-manufacturer tenders, will limit competition in biosimilars [16]. This paper was considered by the panel, together with additional evidence summarized in Appendix C.

## 4. Discussion

A Delphi process, involving diverse stakeholders from across Europe, was conducted to achieve a consensus opinion on biosimilar market sustainability in Europe. Divergent views between stakeholder groups, and the reasons for these, were explored through individual, anonymized feedback and facilitated discussion at a roundtable meeting. This important exercise was undertaken to increase our understanding of the current system and to address concerns regarding sustainability, including the unmet need to develop a long-term vision, as highlighted in previous analyses [11,19,20]. Participants agreed that a sustainable biosimilar market must deliver tangible and transparent benefits to the health care system, while meeting the needs of all stakeholders. The definition (as shown in Box 1) was approved by all participants; however, different stakeholder groups emphasized different priorities within this definition, which is consistent with the previous literature on a lack of a unified approach [19,20]. Participants also agreed that, to make this approach work, collaboration between stakeholders is required and a greater awareness of the drivers of and threats to a sustainable market. In brief, strategies around competition, incentives, and procurement policies were identified and discussed with key consensus highlighted in the tables. These areas (notably the need to establish healthy competition, pricing, and market access policies (considering gain sharing and price reductions), government policy and guidance, identification of risks associated with biosimilar drug supply (e.g., quality issues), and patient access to information and education) were highlighted in the previous literature as key areas requiring further improvements [11,19]. Participants in the Delphi process agreed that these key findings should be developed further into a white paper that highlights the need for multistakeholder collaboration on establishing principles for biosimilar procurement in Europe.

Several priorities for future research were identified by stakeholders. First, understanding and measuring the impact of biosimilar transition on hospital and health care services will better enable costs and benefits to be weighed up and help minimize disruption for patients and health care services. Second, there is a need to understand and develop prospective indicators of market sustainability and potential risks to competitive biosimilar markets, particularly the emergence of de facto monopolies and supply risks. Finally, it will also be important to understand the implications of procurement structure and design for biosimilar market sustainability, especially with regard to how the procurement level (national vs. subnational), market size, number of awarded contracts (and market share awarded), and tender criteria affect market sustainability.

There is currently very limited published evidence available to support detailed arguments in the three priority areas described above, largely because there are limited data with which to conduct analyses. Biosimilar markets are still relatively new in Europe, which means that the available data relate to limited time periods and newly emerging trends that may be expected to mature over time. Further, the currently available data (e.g., on supply shortages of biosimilars) are kept at the national level; this allows cross-country comparisons but poses a challenge for pan-European analysis. It is therefore recommended that any further research begins with a scoping phase, in which the available data are reviewed in detail to assess their suitability for the proposed purpose. Further research would also benefit from a more quantifiable approach to the sustainability framework, allowing us to measure the extent to which a biosimilar market in a specific jurisdiction can be effectively maintained.

Collaboration with stakeholders to develop principles for biosimilar procurement may be progressed in tandem with further research. The objective of establishing processes is to ensure that the concerns of all stakeholders—patients, physicians, pharmacists, payers, policy advisers, and manufacturers—are considered in procurement design. In the absence of evidence, open communication and collaboration between stakeholders may provide the necessary information that procurement decision makers need to prevent risks to biosimilar market sustainability from materializing.

This Delphi process involved a limited number of stakeholders and, as with any Delphi exercise, may also be biased by those who chose to participate [31]. For example, a number of issues were not considered such as the evolution of the biosimilar production process over time. However, the process encompassed evidence from a broad review of available literature and covered a broad range of stakeholder perspectives. Despite a rigorous approach, the findings of the SLR indicated that there was an absence of consistent, comprehensive information about drug shortages (specifically biosimilar shortages) and the costs of switching to biosimilars in Europe; these gaps exacerbate a lack of evidence regarding the impact of different tender models for savings, sustainable competition, and supply risk. The panel identified eight key papers (Appendix C), some of which were not identified by the SLR. The consensus reached by the Delphi process provides further direction for future research into, and implementation of, potential strategies to support these different aspects of sustainability.

## 5. Conclusions

A sustainable biologics market including biosimilars is essential for ensuring that health care savings are maintained into the future, both for existing molecules and those approaching a loss of exclusivity. This Delphi approach resulted in a consensus definition of biosimilar market sustainability in Europe, specified the components of a sustainable biosimilar market, and identified key drivers and risks to sustainability. Crucially, participants in the Delphi process highlighted the need for multistakeholder collaboration in designing policy and practice relating to biosimilars (including procurement). Further research is required alongside stakeholder collaboration to inform biosimilar policy and practice in alignment with the principles identified in this Delphi process. Failure to care for biosimilar market sustainability may impoverish the biosimilar development and offerings, eventually leading to increased cost for health care systems and patients, with fewer resources for innovation.

## Figures and Tables

**Table 1:** Consensus on components of a sustainable biosimilar market.

**I.** **A sustainable biosimilar market must deliver tangible and transparent benefits to the health care system**	
Biosimilars have the potential to reduce the cost of treatment; this, in turn, strengthens the sustainability of health care expenditure	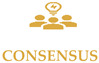
Biosimilar-related savings must be tangible and transparent and should be reinvested efficiently; this may include addressing deficits, and funding innovative therapies, health care or other public services. Biosimilars have the potential to expand access	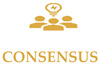
Providers (physicians and pharmacists) incur real costs when transitioning to a new biosimilar; transition should only occur if savings substantially exceed these transition costs and a portion of the savings are used to meet these costs	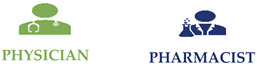
**II.** **A sustainable biosimilar market must address the needs of all stakeholders**	
Transitioning between biosimilars causes disruption to patient care and health care services. Unnecessary disruptions (i.e., frequent transitions and/or transitions that do not deliver tangible savings) should be minimized	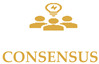
Disruption caused by biosimilar transition may be unavoidable in some therapeutic areas (e.g., acute vs. chronic conditions); however, switch is not advisable if treatment duration is short	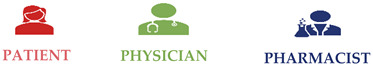
Disruption and transition costs occur in both hospital and out-of-hospital (including retail and home care) settings; these differences may need to be considered	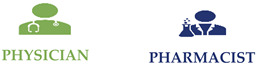
**III.** **A sustainable biosimilar market requires collaboration between stakeholders**	
Policies and practices must encourage trust in biosimilar use among patients through effective communication between stakeholders	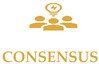
Language and messaging should be consistent among stakeholders and coordinated nationally	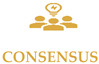
Clear guidance from regulators and clinical organisations at European and national levels is required to motivate multiple switches (i.e., following the initial transition from original biological to biosimilar)	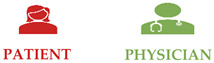
This guidance may benefit from real-world studies (e.g., registry studies)–although not all stakeholders agree that this would be sufficient evidence	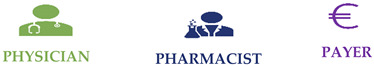
Research would need to be led by providers (pharmacists and physicians), as there are limited incentives for manufacturers to invest in this research	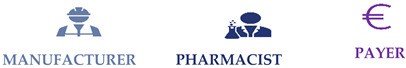

Note: icons shown on the right represent level of agreement between the stakeholders. The ‘consensus’ icon indicates that all stakeholders (physicians, payers, policy advisors, manufacturers, pharmacists, and patients) agreed on that point. Benefits, such as expanded access, have also been noted in the literature [9].

**Table 2:** Consensus on drivers and risks to biosimilar market sustainability (competition and incentives).

**I.** **Competition is a more effective mechanism to achieve a long-term predictable price level than regulation**	
Increased competition leads to more rapid price reduction and, if procurement policies contribute to business continuity, a sustained lower price level	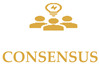
There is a need to develop better prospective indicators to warn about potential risk of *de facto* monopoly	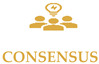
Existing indicators, such as the number of manufacturers and manufacturing sites for biosimilars, are imperfect and may only indicate a problem that is too late to reverse Additional indicators that could be explored include procurement design (e.g., contract length), geographic division (national vs. regional) and factors other than cost	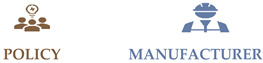
New entrants may bring minor improvements (e.g., administration devices), although competition has been primarily price-focused and has led to a reduction in “value-add” (e.g., patient support programs)	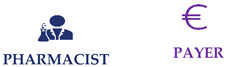
Price-setting regulation, if needed to prevent predatory behaviour, should not aim primarily at the lowest possible prices but at long-term viability of a vibrant and competitive marketplace	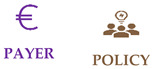
**II.** **There needs to be incentives for investment in future biosimilars**	
Continued investment in biosimilar development and market entry is important to generate competition for biological therapies for which no biosimilar is currently available and, to a lesser extent, therapies with biosimilars already available	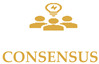
Price expectations of policy and budget holders must reflect market opportunity, e.g., biosimilars of orphan therapies may require lower price discount levels	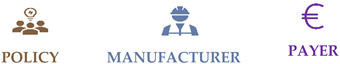
A stable, predictable price level enables manufacturers to make the long-term decisions that are required to invest in biosimilar development	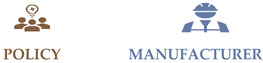
**III.** **Governments and pricing bodies need to drive incentives**	
These bodies need to supply incentives that enable enough suppliers to survive free market onslaught; this may assure the continuity of long-term competition and sustainable discounts from originator biological therapy price levels	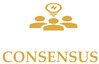
This could be achieved by varying tender available to manufacturers	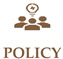

Note: icons shown on the right represent level of agreement between the stakeholders. The ‘consensus’ icon indicates that all stakeholders (physicians, payers, policy advisors, manufacturers, pharmacists, and patients) agreed on that point.

**Table 3:** Consensus on drivers and risks to biosimilar market sustainability (procurement processes).

**I.** **Procurement processes should avoid monopolies and minimize patient and health care system disruption**	
The emergence of monopolies may lead to higher price levels and/or enhanced supply risks	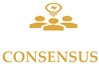
There are examples of this in generics, although these issues would be more pronounced for biosimilars due to lengthy development and market entry processes	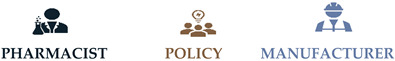
Procurement design should aim to:	
Prevent predatory behaviour, e.g., by considering factors other than price to avoid aggressive price discounting	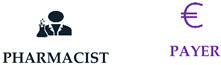
Minimize disruption of patient care, based on the needs of individual therapeutic areas, e.g., by setting contract duration that is proportional to duration of treatment	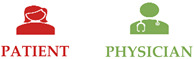
**II.** **The principles for procurement should be agreed by all stakeholders**	
There should be a multistakeholder group that sets principles for policy and practice around biosimilar procurement	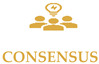
Patients and physicians should have an opportunity for their views to be represented (e.g., in a national forum) and patients should be informed of the rationale behind procurement decisions that impact on their care	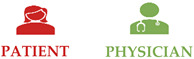
There can be no one-size-fits-all approach to procurement, as the structure and characteristics of health care systems vary; however, there should be a consistent approach and a common set of guiding principles	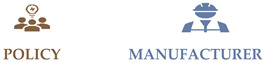

Note: icons shown on the right represent level of agreement between the stakeholders. The ‘consensus’ icon indicates that all stakeholders (physicians, payers, policy advisors, manufacturers, pharmacists, and patients) agreed on that point.

**Figure A1:**
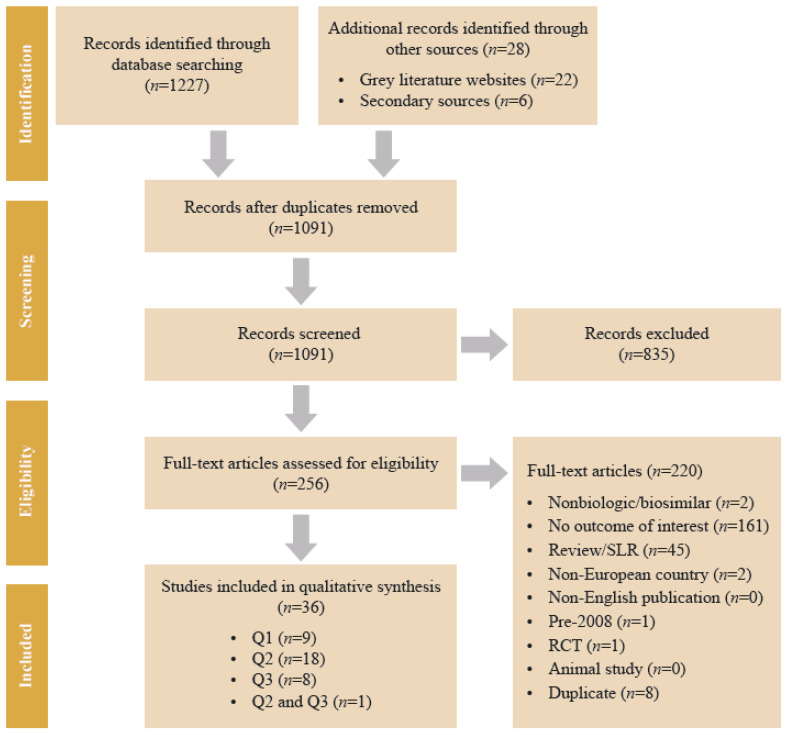
PRISMA Flow Diagram. RCT, randomized controlled trial. SLR, systematic literature review.

## Appendix A

**Table A1:** Brainstorming questionnaire (Delphi process stage 1).

**I.** **Define a sustainable biosimilar market in Europe from your perspective.**	
	What does a sustainable biosimilar market look like? Provide up to five key points
**II.** **What factors contribute to, or create a risk to, biosimilar market sustainability?**	
	How important are each of these factors in achieving sustainability? Provide up to five key points and indicate how important they are to you (low/moderate/high)
**III.** **How have biosimilars contributed to the sustainability of health care system(s)?**	
	Are you aware of any specific, direct benefits from biosimilars entering the market?

## Appendix C

**Table A2:** Key literature identified during brainstorming (Delphi process stage 1).

Topic	Source	References
Savings (implications for health system sustainability)	Systematic literature review/targeted additional search	Vulto A, et al. (2019) [18]
Sustainable competition	Systematic literature review	Mestre-Ferrandiz J, et al. (2016) [16] Dave CV, et al. (2017) [25] Dave CV, et al. (2018) [26]
Access and pricing	Targeted additional search	Moorkens E, et al. (2017) [17] Kawalec P, et al. (2017) [27]
Procurement/purchasing	Review of tender documents (2018)	Vulto A, et al. (2019) [18]
Patient safety/use	Targeted additional search	Tabernero J, et al. (2016) [4] EULAR PARE (2018) [3]

## Appendix D

**Table A3:** Themes and statements (Delphi process stage 2).

Presence of multiple suppliers on an ongoing basis—although there is no “correct” number of suppliers	1. Biosimilars offer choice and are an important savings opportunity that can benefit the health care system broadly
Competition that is effective in reducing prices for biologics/biosimilars to a sustainable level	2. The presence of multiple suppliers offers benefits (with some trade-offs)
Shared decision making with payer, pharmacist, physician, and patients around biosimilar use	3. Competition offers benefits (with some trade-offs)
Reliable supply of biosimilars that meet appropriate standards for quality	4. Effective implementation is necessary for sustainability
Stability in procurement structure and approach	5. Excessive levels of competition have potential to be detrimental to the future of competition in biosimilars markets
Avoidance of price erosion that leads to market exit and the emergence of monopolies or the consolidation of suppliers	6. The level at which procurement happens has consequences for the number of manufacturers in the market—national versus sub-national
